# Effects of Tea against Alcoholic Fatty Liver Disease by Modulating Gut Microbiota in Chronic Alcohol-Exposed Mice

**DOI:** 10.3390/foods10061232

**Published:** 2021-05-28

**Authors:** Bangyan Li, Qianqian Mao, Dandan Zhou, Min Luo, Renyou Gan, Hangyu Li, Siyu Huang, Adila Saimaiti, Ao Shang, Huabin Li

**Affiliations:** 1 Guangdong Provincial Key Laboratory of Food, Nutrition and Health, Department of Nutrition, School of Public Health, Sun Yat-Sen University, Guangzhou 510080, China; liby35@mail2.sysu.edu.cn (B.L.); maoqq@mail2.sysu.edu.cn (Q.M.); zhoudd6@mail2.sysu.edu.cn (D.Z.); luom65@mail2.sysu.edu.cn (M.L.); lihy277@mail2.sysu.edu.cn (H.L.); huangsy9@mail2.sysu.edu.cn (S.H.); saimaiti@mail2.sysu.edu.cn (A.S.); shangao@mail2.sysu.edu.cn (A.S.); 2 Research Center for Plants and Human Health, Institute of Urban Agriculture, Chinese Academy of Agricultural Sciences, Chengdu 610213, China; ganrenyou@caas.cn; 3 Key Laboratory of Coarse Cereal Processing (Ministry of Agriculture and Rural Affairs), Sichuan Engineering & Technology Research Center of Coarse Cereal Industralization, Chengdu University, Chengdu 610106, China

**Keywords:** tea, alcoholic fatty liver disease, inflammation, oxidative stress, gut microbiota

## Abstract

Gut microbiota dysbiosis has been a crucial contributor to the pathogenesis of alcoholic fatty liver disease (AFLD). Tea is a popular beverage worldwide and exerts antioxidant and anti-inflammatory activities, as well as hepatoprotective effects. However, the potential role of gut microbiota regulated by tea in the prevention and management of AFLD remains unclear. Here, the protective effects of oolong tea, black tea, and dark tea on AFLD and its regulation of gut microbiota in chronic alcohol-exposed mice were explored and investigated. The results revealed that tea supplementation significantly prevented liver steatosis, decreased oxidative stress and inflammation, and modulated gut microbiota in chronic alcohol-exposed mice, especially oolong tea and dark tea. However, black tea showed less effectiveness against liver injury caused by alcohol. Moreover, the diversity, structure and composition of chronic alcohol-disrupted gut microbiota were restored by the supplementation of oolong tea and dark tea based on the analysis of gut microbiota. Furthermore, the relationship between liver injury biochemical indicators and gut microbiota indicated that some specific bacteria, such as *Bacteroides*, *Alloprevotella*, and *Parabacteroides* were closely associated with AFLD. In addition, the phytochemical components in tea extracts were measured by high-performance liquid chromatography, which could contribute to preventive effects on AFLD. In summary, oolong tea and dark tea could prevent chronic alcohol exposure-induced AFLD by modulating gut microbiota.

## 1. Introduction

Alcoholic liver disease (ALD) induced by consistently excessive alcohol consumption exhibits extremely high morbidity and mortality as a worldwide epidemic [1]. ALD includes a histological spectrum of liver injury ranging from asymptomatic liver steatosis characterized by abnormal accumulation of triglyceride in hepatocytes to alcoholic hepatitis, with potential progression to fibrosis, cirrhosis, and even hepatic carcinoma [2]. Thus, alcoholic fatty liver disease (AFLD) is the earliest pathological process of ALD and has been thought to play a preeminent role in the reversible pathological stage of ALD [3]. So far, it has been recognized that the best treatment for AFLD is still abstinence from alcohol, and there are few breakthroughs in other areas [4,5]. Therefore, novel and more practical methods for the prevention and management of AFLD have urgently attracted substantial attention.

Up to now, although the molecular mechanism involved in AFLD has not yet been well studied, mounting evidence has documented that multiple factors participate in the pathogenesis of AFLD, such as oxidative stress, inflammation, and gut microbiota dysbiosis [6,7,8,9]. However, more and more scientific studies are performed to support the role of modifiable factors in the initiation and development of ALD, especially the gut microbiota [10,11]. Under normal physiological conditions, the composition of gut microbiota is considered to remain in a symbiotic balance in human body, while that balance can be destroyed by long-term excessive alcohol intake [12]. Considerable research has demonstrated that chronic alcohol exposure causes alterations in the composition of gut microbiota, thereby promoting the overgrowth of a few pathogenic bacteria, inhibiting the dominant gut bacteria, and significantly increasing harmful metabolites (lipopolysaccharide, LPS) entering the liver [13,14,15]. For instance, a significant increase in the abundance of *Actinobacteria* and *Proteobacteria*, and an obvious reduction in that of *Firmicutes* and *Bacteroidetes* are observed among mice exposed to alcohol [14]. Additionally, an animal study suggested that there is no obvious damage in liver among germ-free mice exposed to alcohol, which indicated that alcohol exposure alone is not enough to cause the development of liver disease [16]. On the other hand, the composition of disordered gut microbiota induced by alcohol exposure could be restored to close to that of alcohol-resistant mice through fecal microbiota transplantation experiment, which also revealed that gut microbiota could play a crucial role in the occurrence and development of AFLD [17]. Moreover, a previous study showed that the LPS ectopic to the liver could bind to Toll-like receptor 4 (TLR-4) and activate nuclear factor-κB (NF-κB) pathway, which promoted the release of inflammatory cytokines and excessive reactive oxygen species (ROS) [18]. Thus, targeting intestinal microbiota to prevent and manage the occurrence and progression of AFLD may be an effective therapeutic strategy, and relative studies are needed urgently.

Tea made from the leaves of the plant *Camellia sinensis* is one of the most widely consumed functional beverages in the global population [19]. Numerous studies have proven that tea exhibits various health-promoting effects, including antioxidant capacity, protection against cancer, and anti-obesity and hepatoprotective effects [20,21,22,23,24]. In addition, it has been found that these biological activities are mainly attributed to the major functional components, such as polyphenols [25]. Depending on the different fermentation degrees and processes, tea can be generally classified into six major types, including green tea, yellow tea, white tea, oolong tea, black tea, and dark tea [26]. The green tea, yellow tea, and white tea were un-fermented or slight-fermented. The oolong tea (semi-fermented), black tea (deep-fermented) and dark tea (post-fermented) have received increasing attention on the regulation of gut microbiota in recent years, because many microbes and metabolites were produced in the fermented process [27,28,29,30]. For example, a previous study has reported that black and oolong teas could significantly change the abundance of *Bacteroides*, *Parabacteroides*, and *Akkermansia* in mice fed with a high-fat diet (HFD) [31]. Similarly, another research has shown that Fuzhuan Brick Tea, one dark tea, could alter the structure of gut microbiota community in HFD-fed mice, with a significant increase in the abundance of *Bifidobacteriaceae* [32]. However, the potential role of gut microbiota modulated by oolong tea, black tea and dark tea in the prevention and management of AFLD is still not clearly understood. Therefore, in the present study, our aim is to explore and investigate the effects of oolong tea, black tea, and dark tea on AFLD in chronic alcohol-exposed mice and the potential role of gut microbiota in mediating the effects of these teas on AFLD.

## 2. Materials and Methods

### 2.1. Preparation of Tea Extracts

The specific information about the two black teas, two oolong teas, and two dark teas purchased from China is shown in Table 1. The preparation method of the tea extracts was as follows. Briefly, the tea sample (10 g) was extracted three times with 100 mL of boiling water in water bath shaker-DKZ-450B (98 °C) (Sensin, Shanghai, China) for 10 min. Subsequently, all extracted solutions were combined and filtered, and then concentrated via a rotary evaporator (R-501, Cancun, Shanghai, China) under a vacuum environment. Lastly, the concentrated filtrate from tea extract was freeze-dried through a freeze dryer (Labconco-7752001, Kansas City, MO, USA) to obtain a powder, which was stored at −80 °C.

### 2.2. Experimental Animals and Feeding

The 8-week-old C57BL/6J male mice were used in this research, which were purchased from the Guangdong Medical Laboratory Animal Center (Guangzhou, China). All mice were fed in specific-pathogen-free (SPF) laboratory animal room and they could eat and drink freely. Additionally, the animal room was maintained under the light (12 h light–dark cycle), the relative humidity (40–60%) and the temperature (22 ± 0.5 °C) controlled environment. Moreover, the AFLD mouse models were induced by the modified Lieber-DeCarli ethanol liquid diet (TP 4030B), and animals in the control group were fed with a Lieber-DeCarli control liquid diet (TP 4030C), which was purchased from TROPHIC Animal Feed High-tech Co., Ltd. (Nantong, China). The energy composition of carbohydrates, protein and fat were 19%, 18%, and 35%, respectively, from the Lieber-DeCarli ethanol liquid diet, in which ethanol supplied 28% of total calories. However, the energy supply of ethanol was substituted by carbohydrates in the Lieber-DeCarli control liquid diet. Furthermore, the 95% ethanol was obtained from Guangzhou Wego Instrument Co., Ltd. (Guangzhou, China).

### 2.3. Experimental Design

The AFLD mouse model was established based on the method described in a previous study [33]. Firstly, all experimental mice were fed with the Lieber-DeCarli control liquid diet for 5 days *ad libitum*. Subsequently, mice were randomly divided into ethanol-fed and control groups according to body weight. The mixture of Lieber-DeCarli ethanol and control liquid diets at the ratio of 1:2, 1:1, and 2:1 was treated to ethanol-fed groups, while the control group received the Lieber-DeCarli control liquid diet. Then, after 6 days, the ethanol-fed mice were further divided into several groups (9 mice per group), including a model group and six tea extract supplementary groups, which were fed with the Lieber-DeCarli ethanol liquid diet containing 4% (*w*/*v*) ethanol. Due to the particularity of liquid feed, the mice were divided into small cages, 3 in each cage. In addition, tea extract supplementary groups were administered with different tea extracts including Dianhong Tea (BT1), Yingde Black Tea (BT2), Tieguanyin Tea (OT1), Fenghuang Danzong Tea (OT2), Fu Brick Tea (DT1), and Selenium-Enriched Dark Tea (DT2) at the dose of 200 mg/kg b.w. for 4 weeks, while the model and control groups were given distilled water (10 mL/kg) by gavage for 4 weeks. Afterwards, fecal samples of each mouse were collected, and then stored at −80 °C until processing. Then, all the mice of different groups fasting for 9 h were weighed, anesthetized, and sacrificed to collect blood and liver samples used for further experiments. All experimental procedures involving animals in this study were approved by “Principles of Care and Use of Laboratory Animals” at the School of Public Health, Sun Yat-Sen University (approval number: 2019-002; 28 February 2019).

### 2.4. Serum TG, TC Levels, and Aminotransferase Activities Measurement

After the collected blood sample kept at room temperature for one hour, the serum sample was obtained by centrifugation at 4000× *g* at 4 °C for 10 min. Subsequently, the activities of serum aspartate transaminase (AST) and alanine transaminase (ALT), and the levels of serum total cholesterol (TC) and triglyceride (TG) in each group were, respectively, determined by the automated biochemistry analyzer (Roche, Mannheim, Germany). In addition, the kits of serum ALT, AST, TG, and TC were purchased from Roche diagnostics (Shanghai, China).

### 2.5. Hepatic Tissue Staining

After mice were sacrificed, the liver tissues were removed immediately and fixed with 4% paraformaldehyde for two days. The liver histological analysis was performed to evaluate liver injury including hepatocyte lipid accumulation change, the infiltration of inflammatory cells and degeneration using Hematoxylin-eosin (H&E) staining. The liver samples were embedded in paraffin and processed to prepare 5 µm paraffin sections for Hematoxylin-eosin (H&E) staining. Moreover, the images were captured via a light microscope (Leica, Solms, Germany).

### 2.6. Biochemical Analysis of Hepatic Tissue

The detection kits of hepatic glutathione peroxidase (GSH-Px), glutathione (GSH), superoxide dismutase (SOD), catalase (CAT), alcohol dehydrogenase (ADH), and acetaldehy dehydrogenase (ALDH) were gained from Nanjing Jiancheng Bioengineering Institute (Nanjing, China). Afterward, liver samples were made by homogenizing in physiological saline solution (0.9%). Then, the hepatic homogenate was centrifuged (2500× *g*, 10 min, and 4 °C) to gain the supernatant, which was taken for the determination of GSH-Px, SOD, CAT, and ADH activities, and the GSH content according to the instructions. Specially, the supernatant used to analyze the activity of ALDH was needed to be centrifuged at 10,000× *g* for 10 min at 4 °C.

On the other hand, the determination kits of hepatic TG, TC, malondialdehyde (MDA), and total protein were purchased from Apply-gen Technologies Inc. (Beijing, China). The 25 mg of frozen liver tissue was mixed with 500 μL of lysis buffer, and incubated at 4 °C for 30 min, and then was ground with TissueLyser II Qiagen (QIAGEN^®^, Hilden, Germany). Subsequently, the liver homogenate was heated at 70 °C for 10 min and centrifuged at 2000× *g* at 4 °C for 5 min to gain the supernatant, which was used to analyze hepatic TG, TC, and total protein concentrations. Specially, the supernatant used for the detection of MDA content was obtained after the liver homogenate was centrifuged for 10 min at 10,000× *g* at 4 °C based on the manufacturer’s instructions.

### 2.7. Enzyme-Linked Immunosorbent Assay (ELISA)

The liver samples were made via homogenization in sterile PBS. Then, the homogenates were centrifuged at 3000× *g* for 20 min at 4 °C to harvest the supernatants, which were used for the determination of cytochrome P450 2E1 (CYP2E1), 4-hydroxynonenoic acid (4-HNE), tumor necrosis factor-α (TNF-α), and interleukin-6 (IL-6) using ELISA Kits (Meimian, Jiangsu, China) [34,35].

### 2.8. Measurement of Phytochemicals in Teas

The phytochemical components in tea extracts were detected using high-performance liquid chromatography (HPLC) with the standard compounds from Derick Biotechnology Co., Ltd. (Chengdu, China) according to our previous report [36].

### 2.9. Gut Microbiota Analysis

After 4 weeks of intervention, mice were transferred to a freshly sterilized cage. Subsequently, the fresh and uncontaminated (by urine) fecal samples of each mouse were collected separately in sterilized EP tubes, and frozen immediately in the liquid nitrogen. Then, the EP tubes were stored in a refrigerator at −80 °C until further processing. In addition, the information of microbial composition and structure in collected fecal samples was analyzed by 16S rRNA gene sequencing [37,38].

#### 2.9.1. Fecal Sample DNA Extraction

Total microbial genomic DNA was extracted from the fecal sample using ALFA-SEQ Advanced Stool DNA Kit (Magi gene, Guangdong, China) based on the instruction of the kit. After that, the extracted DNA concentration was measured by Thermo Nano Drop One (Thermo Fisher, Waltham, MA, USA, and the 1% agarose gel electrophoresis was used to measure the integrity and purity of the DNA sample. The DNA samples were then stored in a refrigerator at −20 °C before further study.

#### 2.9.2. Amplification and Sequencing of the 16S rRNA Genes

The 16S rRNA V3+V4 hypervariable region was amplified by polymerase chain reaction (PCR) using the forward primer 5′-ACTCCTACGGGAGGCAGCA-3′ and reverse primer 5′-GGACTACHVGGGTWTCTAAT-3′. In addition, the program of PCR amplification was carried out with DNA polymerase in a thermocycler and was started from pre-denaturation at the condition of 94 °C for 5 min. After 31 cycles including denaturation at 94 °C for 30 s, annealing at 52 °C for 30 s, and extension at 72 °C for 45 s, the program finally stopped after an extension of 10 min at 72 °C. The concentrations of PCR products were compared by Gene Tool Analysis Software (Version 4.03.05.0, SynGene, Frederick, MD, USA). Subsequently, the required volume of each sample was calculated based on the principle of equal mass, and the PCR products were mixed. Afterward, the mixed PCR products were recovered by E.Z.N.A. TM Gel Extraction Kits (Omega, Norcross, GA, USA), and the target DNA fragment was eluted with TE buffer. Moreover, the quality of the recovered target DNA fragment was detected via 1% agarose gel electrophoresis and ensured the DNA concentration to be sequenced more than 60 ng/µL. Finally, the target DNA fragment was sequenced at Guangzhou Magigene Technology Co., Ltd. (Guangzhou, China), using the Illumina Novaseq 6000 sequencing platform.

### 2.10. Bioinformatics and Statistical Analysis

All experimental data were presented as mean ± standard deviation. The statistical analysis between groups was performed by SPSS software 20.0 (IBM SPSS Statistics, IBM Corp, Somers, NY, USA), and comparisons among groups were analyzed using least significant difference (LSD) tests. In addition, the *p*-value < 0.05 was considered statistically significant. Moreover, the graphs were produced using GraphPad Prism 8 software (GraphPad Software, La Jolla, CA, USA).

All the original data of the gut microbiota information from fecal sample were cleaned and filtered by using quantitative insights into microbial ecology (QIIME) (http://qiime.org/, accessed on 10 March 2021) o obtain the high-quality clean labels. Subsequently, all sequences with 97% identity as a threshold were clustered to generate an operational taxonomic unit (OUT). Firstly, alpha diversity was conducted for the comparison of microbial community richness (Chao 1’s index) and diversity (Shannon’s and Simpson’s index) among all experimental groups. In addition, the differences of microbial community structure and composition in individuals were performed by beta-diversity analysis based on principal coordinate analysis (PCoA) using R software (Version 3.1.0). Furthermore, linear discriminant analysis effect size analysis (LEfSe) was performed to analyze the most differentially abundant taxa in gut microbiota from phylum to genus and show the statistical significance of the differentially abundant taxa represented in cladograms based on (LDA) score > 4.

## 3. Results and Discussions

### 3.1. Effects of Tea Extracts on Liver Coefficient, Hepatic TG Level, and Body Weight

**Figure 1:**
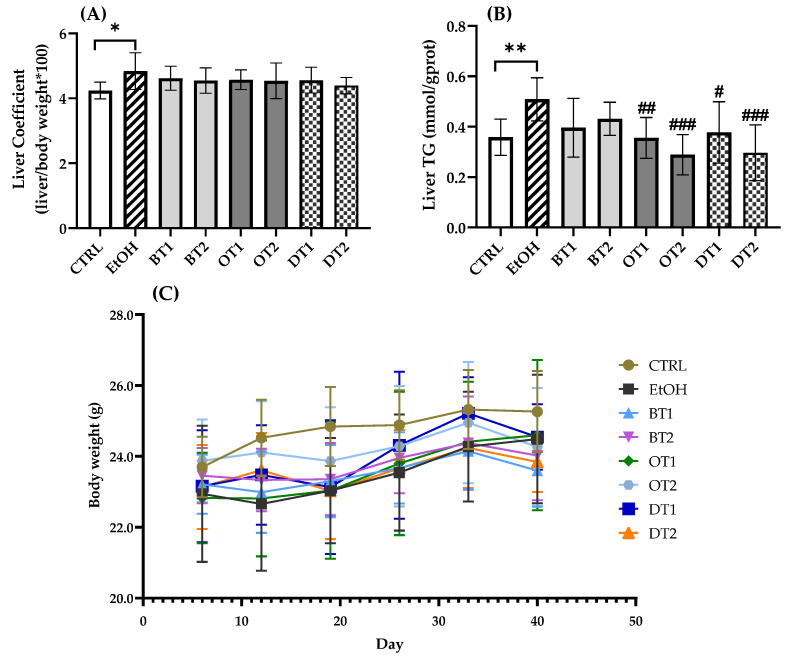
The effects of teas on (**A**) liver coefficient, (**B**) hepatic TG level and (**C**) body weight in mice exposed to chronic alcohol consumption. CTRL, the control group; EtOH, the model group; BT1, Dianhong Tea; BT2, Yingde Black Tea; OT1, Tieguanyin Tea; OT2, Fenghuang Danzong Tea; DT1, Fu Brick Tea; DT2, Selenium-Enriched Dark Tea. * *p* < 0.05, ** *p* < 0.01, the model group compared with the control group; # *p* < 0.05, ## *p* < 0.01, ### *p* < 0.001, the tea extract supplementary groups compared with the model group.

The effects of two black teas, two oolong teas, and two dark teas on liver coefficient, hepatic TG level, and body weight in mice exposed to long-term excessive alcohol consumption are displayed in Figure 1. Mounting evidence demonstrated that chronic alcohol exposure caused abnormal lipid accumulation and hepatic steatosis, resulting in liver injury [39]. The model group showed an obvious increase in liver coefficient (*p* < 0.05) and hepatic TG level (*p* < 0.01) compared with the control group. However, there was no marked difference in body weight among the model, control, and all tea extract supplementary groups, as shown in Figure 1C (*p* > 0.05). In addition, although a decreased tendency in liver coefficient was observed in all tea extract supplementary groups in comparison with the model group, there was no marked difference in Figure 1A (*p* > 0.05). As seen from Figure 1B, the administration of Tieguanyin Tea (OT1), Fenghuang Danzong Tea (OT2), Fu Brick Tea (DT1), and Selenium-Enriched Dark Tea (DT2) extracts significantly inhibited the increase in hepatic TG level induced by chronic alcohol consumption. However, Dianhong Tea (BT1) and Yingde Black Tea (BT2) did not significantly inhibit the hepatic TG accumulation.

### 3.2. Effects of Tea Extracts on Aminotransferase Activities and Serum TG and TC Levels

**Figure 2:**
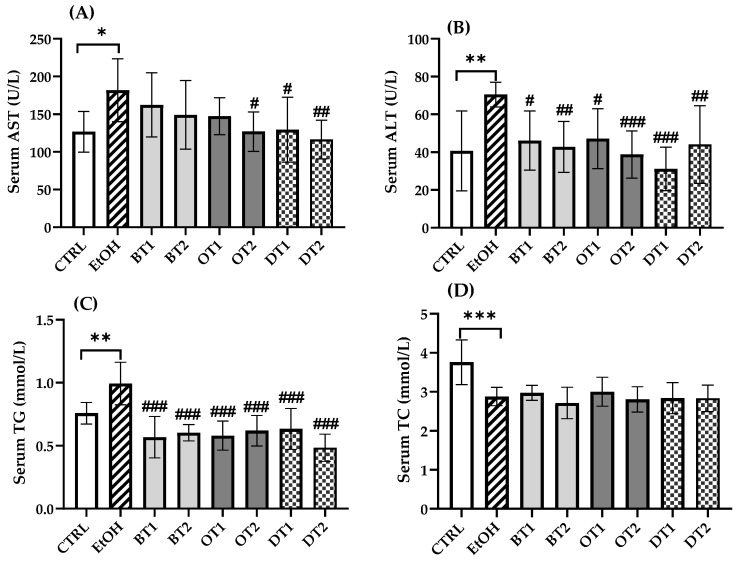
The effects of teas on serum biochemical markers in mice exposed to chronic alcohol consumption. (**A**) AST, aspartate transaminase; (**B**) ALT, alanine aminotransferase; (**C**) TG, triacylglycerol; (**D**) TC, total cholesterol. CTRL, the control group; EtOH, the model group; BT1, Dianhong Tea; BT2, Yingde Black Tea; OT1, Tieguanyin Tea; OT2, Fenghuang Danzong Tea; DT1, Fu Brick Tea; DT2, Selenium-Enriched Dark Tea. * *p* < 0.05, ** *p* < 0.01, *** *p* < 0.001, the model group compared with the control group; # *p* < 0.05, ## *p* < 0.01, ### *p* < 0.001, the tea extract supplementary groups compared with the model group.

The elevated AST and ALT levels indicated the increased permeability and damage in liver. As can be seen from Figure 2A,B, the serum AST and ALT levels in model group were significantly increased to 1.4-fold (181.8 ± 39.1 vs. 126.7 ± 25.4) (*p* < 0.05) and 1.7-fold (70.5 ± 6.1 vs. 40.7 ± 19.9) (*p* < 0.01) compared with those in the control group, respectively. In addition, the model group showed an obviously higher serum TG level (*p* < 0.01) and lower serum TC level (*p* < 0.001) than the control group. It has been reported that tea extract contained antioxidant polyphenols and showed hepatoprotective effect against alcohol-caused liver damage in vivo [40]. According to our results, in the treatment groups with Fenghuang Danzong Tea (OT2), Fu Brick Tea (DT1), and Selenium-Enriched Dark Tea (DT2) extracts, the levels of both serum AST and ALT were markedly decreased, while the administration of Dianhong Tea (BT1), Yingde Black Tea (BT2), and Tieguanyin Tea (OT1) extracts only reduced serum ALT level (*p* < 0.05). All tea extract supplementation significantly reduced the serum TG level in comparison with the model group (*p* < 0.001), but there was no significance among the supplementary groups. For another thing, there was no significant difference in serum TC level between all tea extract supplementary groups and the model group (*p* > 0.05).

### 3.3. Histopathological Evaluation

**Figure 3:**
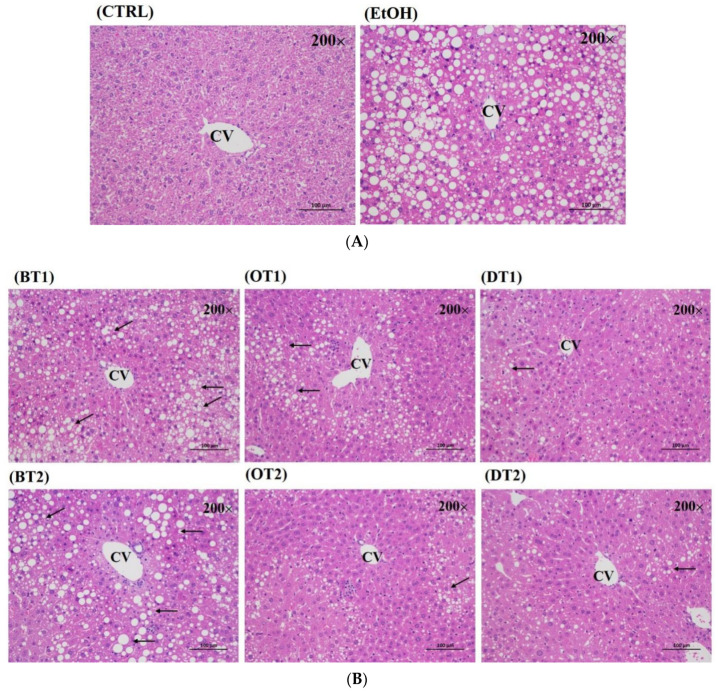
The histopathological examination of hematoxylin and eosin (H&E) stained for all groups (magnification: 200, scale bar: 100 μm). (**A**) CTRL, the control group; EtOH, the model group; (**B**) BT1, Dianhong Tea; BT2, Yingde Black Tea; OT1, Tieguanyin Tea; OT2, Fenghuang Danzong Tea; DT1, Fu Brick Tea; DT2, Selenium-Enriched Dark Tea.

The protective effects of these tea extract supplementation against liver injury induced by chronic consumption of alcohol were further verified by histopathological analysis. As seen from Figure 3A, no obvious pathological abnormality was observed in the control group. However, the hematoxylin-eosin (H&E) staining of the model group showed visible pathological changes, such as a large amount of medium and tiny lipid droplets, disordered cell arrangement and infiltration of inflammatory cells. On the other hand, compared with the model group, Tieguanyin Tea (OT1), Fenghuang Danzong Tea (OT2), Fu Brick Tea (DT1) and Selenium-Enriched Dark Tea (DT2) extract supplementary groups presented less pathological change, which indicated that these teas had stronger preventive effects against fatty liver induced by chronic alcohol consumption. However, the liver tissue in Dianhong Tea (BT1) and Yingde Black Tea (BT2) treatment groups also revealed remarkably pathological damage.

### 3.4. Effects of Tea Extracts on Alcohol Metabolism and Hepatic Lipid Peroxidation Levels

**Figure 4:**
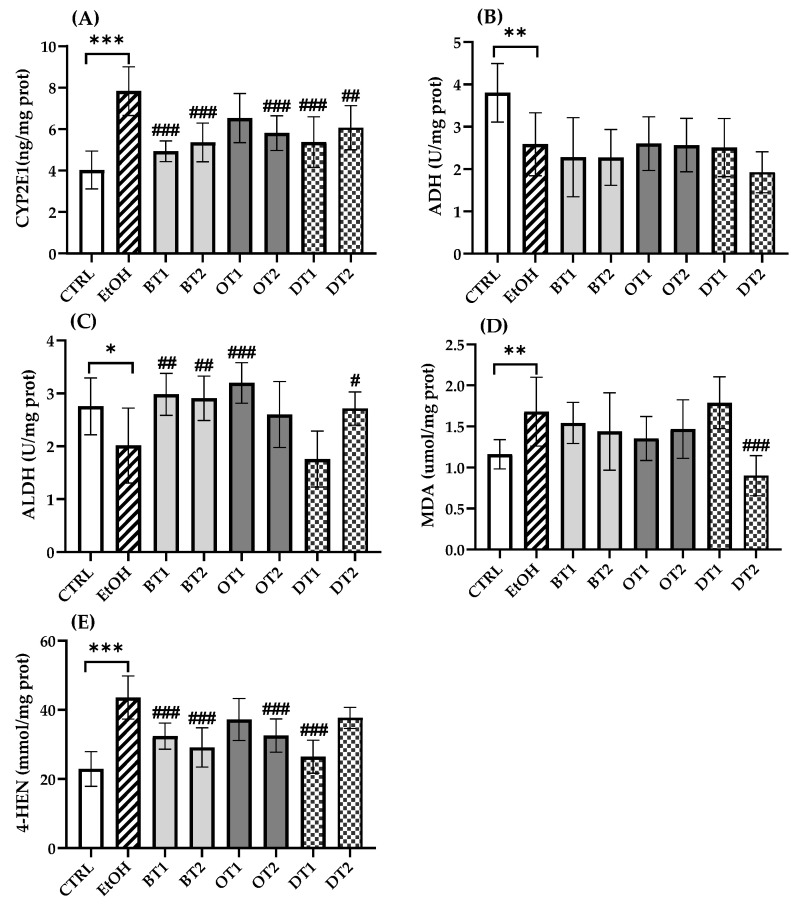
The effects of teas on alcohol metabolism and hepatic lipid peroxidation level in mice exposed to chronic alcohol consumption. (**A**) CYP2E1, cytochrome P450 2E1; (**B**) ADH, alcohol dehydrogenase; (**C**) ALDH, aldehyde dehydrogenase; (**D**) MDA, malondialdehyde; (**E**) 4-HNE, 4-hydroxynonenoic acid. CTRL, the control group; EtOH, the model group; BT1, Dianhong Tea; BT2, Yingde Black Tea; OT1, Tieguanyin Tea; OT2, Fenghuang Danzong Tea; DT1, Fu Brick Tea; DT2, Selenium-Enriched Dark Tea. * *p* < 0.05, ** *p* < 0.01, *** *p* < 0.001, the model group compared with the control group; # *p* < 0.05, ## *p* < 0.01, ### *p* < 0.001, the tea extract supplementary groups compared with the model group.

Chronic alcohol consumption resulted in disturbed alcohol metabolism. It has been proven that more than 90% of ethanol metabolism occurred in the liver [41]. Generally, ADH initially oxidizes ethanol to acetaldehyde, which is subsequently oxidized to acetate via ALDH [42]. In addition, only a little ethanol is oxidized to acetaldehyde via CYP2E1 in physiological conditions [7,43]. However, upon chronic exposure to alcohol, CYP2E1 was induced and produced oxidative stress [44]. As displayed in Figure 4, the expression of CYP2E1 (*p* < 0.001) was significantly elevated and the activities of ADH (*p* < 0.01) and ALDH (*p* < 0.05) were remarkably inhibited in the model group compared with the control group, which was harmful to health [45]. From Figure 4A, the hepatic CYP2E1 expression was significantly decreased in most of the tea extract groups including Dianhong Tea (BT1), Yingde Black Tea (BT2), Fenghuang Danzong Tea (OT2), Fu Brick Tea (DT1), and Selenium-Enriched Dark Tea (DT2). However, the treatment with Tieguanyin Tea (OT1) extract did not significantly inhibit the expression of CYP2E1 (*p* > 0.05). On the other hand, the activity of ALDH was significantly boosted with Dianhong Tea (BT1), Yingde Black Tea (BT2), Tieguanyin Tea (OT1), and Selenium-Enriched Dark Tea (DT2) extracts (*p* < 0.05), which were in accordance with previous research which reported that iced black tea beverages could elevate ALDH activity [46]. Furthermore, our results also suggested that these teas could promote alcohol metabolism to a certain extent. However, there was no marked difference in the activity of ADH between all tea extract treatment groups and the model group (*p* > 0.05).

The increased expression of CYP2E1 would produce a large amount of ROS during alcohol metabolism, leading to severe oxidative stress damage. MDA and 4-HNE are important markers of hepatic lipid peroxidation, and the change in the levels of MDA and 4-HNE can indicate the degree of lipid peroxidation and indirectly reflect liver damage. Figure 4 displays the effects of tea extracts on lipid peroxidation levels in liver tissues, and significant elevation in MDA (*p* < 0.01) and 4-HNE (*p* < 0.001) levels are observed in the model group. The results were consistent with a previous animal study that CYP2E1 expression and lipid peroxidation significantly elevated after long-term excessive alcohol consumption [47]. It has been reported that knockout of CYP2E1 gene or use of CYP2E1 inhibitor thiazole could significantly reduce alcohol-induced oxidative stress and lipid peroxidation [48,49]. Compared with the model group in Figure 4D, only the intervention of Selenium-Enriched Dark Tea (DT2) extract could significantly lower hepatic MDA level (*p* < 0.001). Furthermore, we found that the level of 4-HNE was decreased significantly in four tea extract treatment groups including Dianhong Tea (BT1), Yingde Black Tea (BT2), Fenghuang Danzong Tea (OT2), and Fu Brick Tea (DT1) (*p* < 0.001).

### 3.5. Effects of Tea Extracts on Hepatic Antioxidant Capacity

**Figure 5:**
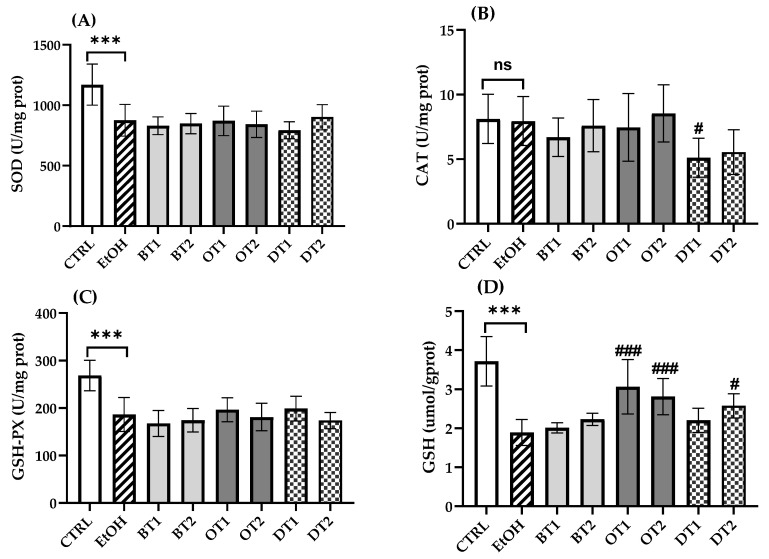
The effects of teas on hepatic antioxidant capacity in mice exposed to chronic alcohol consumption. (**A**) SOD, superoxide dismutase; (**B**) CAT, catalase; (**C**) GSH-Px, glutathione peroxidase; (**D**) GSH, glutathione. CTRL, the control group; EtOH, the model group; BT1, Dianhong Tea; BT2, Yingde Black Tea; OT1, Tieguanyin Tea; OT2, Fenghuang Danzong Tea; DT1, Fu Brick Tea; DT2, Selenium-Enriched Dark Tea. *** *p* < 0.001, the model group compared with the control group; # *p* < 0.05, ### *p* < 0.001, the tea extract supplementary groups compared with the model group.

Under normal physiological conditions, the liver possesses a powerful antioxidant system, which can balance the production of excessive ROS. However, the body’s stable antioxidant system can be destroyed by long-term intake of alcohol. Increasing evidence has demonstrated that acute and chronic ethanol feeding decreased the hepatocyte antioxidant capacity [43,50]. After chronic alcohol feeding, it was found that SOD and GSH-Px activities and GSH content in the mouse liver significantly decreased compared with those antioxidant markers in the control group (*p* < 0.001, Figure 5). However, there was no significant difference in CAT activity between the model and control groups (*p* > 0.05). Similarly, it also showed no significant difference in SOD and GSH-Px activities of all tea extract supplementary groups compared with the model group during chronic ethanol feeding (*p* > 0.05). In addition, the activity of CAT was remarkably inhibited in Fu Brick Tea (DT1) extract supplementary group in comparison with the model group (*p* < 0.05). For another thing, Tieguanyin Tea (OT1), Fenghuang Danzong Tea (OT2) and Selenium-Enriched Dark Tea (DT2) extract supplementary groups showed significantly higher levels of GSH than the model group, suggesting that these teas elevated the antioxidant ability, which was consistent with our previous research [36].

### 3.6. Effects of Tea Extracts on Hepatic Inflammatory Cytokine Levels

**Figure 6:**
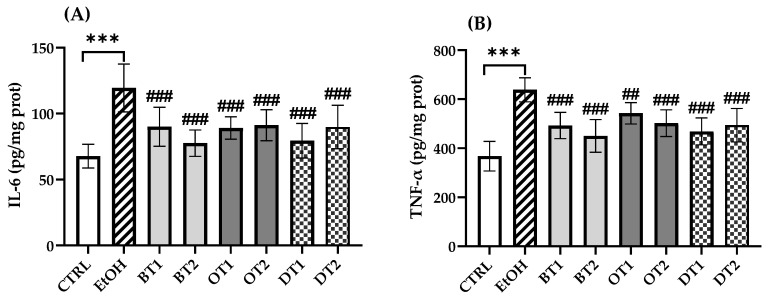
The effects of teas on hepatic inflammatory cytokines levels in mice exposed to chronic alcohol exposure. (**A**) IL-6, interleukin-6; (**B**) TNF-α, tumor necrosis factor-α. CTRL, the control group; EtOH, the model group; BT1, Dianhong Tea; BT2, Yingde Black Tea; OT1, Tieguanyin Tea; OT2, Fenghuang Danzong Tea; DT1, Fu Brick Tea; DT2, Selenium-Enriched Dark Tea. *** *p* < 0.001, the model group compared with the control group; ## *p* < 0.01, ### *p* < 0.001, the tea extract supplementary groups compared with the model group.

In this research, the production of inflammatory cytokines was measured and it was found that IL-6 and TNF-α levels in the model group remarkably elevated in comparison with the control group (*p* < 0.001, Figure 6). Similarly, the levels of IL-6 and TNF-α in the liver tissue significantly decreased in all tea extract supplementary groups compared with the two cytokines in the model group, but there was no significant difference among all the tea extract supplementary groups.

### 3.7. Effects of Tea Extracts on the Diversity and Structure of Gut Microbiota

Mounting evidence has reported that gut microbiota dysbiosis is a major contributor to the initiation and progression of AFLD [51]. Recently, several experimental and clinical studies have reported that alcohol consumption influenced the diversity, structure and composition of gut microbes, and gut microbiota dysbiosis is strongly related to ALD [17,52,53]. Here, the microbial richness, diversity and structure of fecal samples at the end of the experiment were analyzed and compared using Illumina Novaseq 6000 sequencing. As displayed in Table 2, the alpha-diversity analysis revealed that significant differences were observed in the richness and diversity of intestinal microbiota between the model group and the control group, as evidenced by the Chao 1 richness index and the Simpson as well as Shannon diversity index. These data suggest that chronic alcohol exposure can cause bacterial overgrowth and reduce gut microbiota diversity. For another thing, the increased richness and decreased diversity in gut microbiota induced by alcohol exposure were significantly restored after the intervention of Tieguanyin Tea (OT1), Fenghuang Danzong Tea (OT2), Fu Brick Tea (DT1), and Selenium-Enriched Dark Tea (DT2) extracts. However, Dianhong Tea (BT1) and Yingde Black Tea (BT2) extract treatments insignificantly influenced both richness and diversity in gut microbiota compared to the model group.

**Figure 7:**
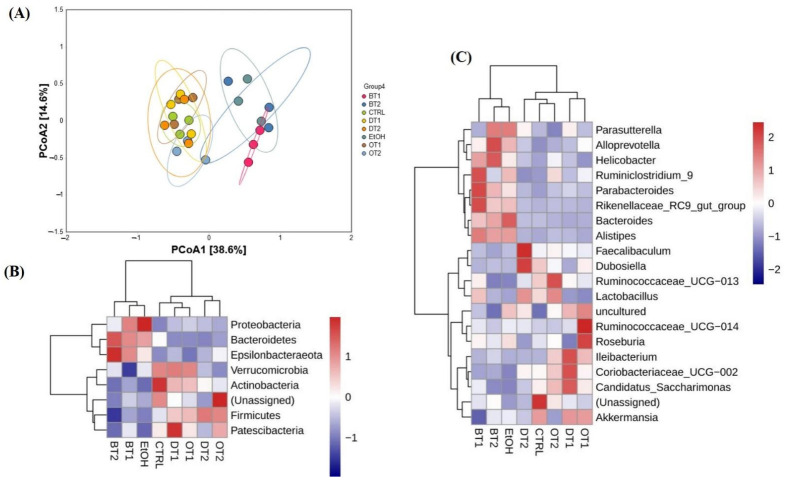
The effects of teas on the structure of microbiota in mice affected by chronic alcohol exposure. (**A**) PCoA analysis. (**B**) species abundance cluster analysis at the phylum level. (**C**) species abundance cluster analysis at the genus level. CTRL, the control group; EtOH, the model group; BT1, Dianhong Tea; BT2, Yingde Black Tea; OT1, Tieguanyin Tea; OT2, Fenghuang Danzong Tea; DT1, Fu Brick Tea; DT2, Selenium-Enriched Dark Tea.

Subsequently, to investigate the similarities of the structure and composition of the gut microbial community among different fecal samples or groups, beta-diversity analysis and species abundance cluster analysis were carried out, respectively. Seen from Figure 7A, beta-diversity analysis based on PCoA analysis indicated that the microbial structure was remarkably disturbed by chronic alcohol exposure compared to the control group, but the influence could be inhibited and remedied to normal status through the treatment of Tieguanyin Tea (OT1), Fenghuang Danzong Tea (OT2), Fu Brick Tea (DT1), and Selenium-Enriched Dark Tea (DT2) extracts. However, the microbial structures in the fecal samples of Dianhong Tea (BT1) and Yingde Black Tea (BT2) groups were in accordance with the model group, and the three groups were clearly separated from other groups along PCoA1 (38.6%). It was also indicated that Dianhong Tea (BT1) and Yingde Black Tea (BT2) extracts did not significantly inhibit the change of microbial structure caused by chronic alcohol intake.

As expected, the results of species abundance cluster analysis based on both phylum and genus levels showed that overt change in the composition of gut microbiota was observed in the model group compared with the control group as displayed in Figure 7B,C. In addition, Tieguanyin Tea (OT1), Fenghuang Danzong Tea (OT2), Fu Brick Tea (DT1), Selenium-Enriched Dark Tea (DT2), and control groups had similar microbial community composition, while the Dianhong Tea (BT1) and Yingde Black Tea (BT2) and model groups were in the same cluster, which was in line with the results of PCoA analysis.

### 3.8. Effects of Tea Extracts on the Composition of Gut Microbiota

**Figure 8:**
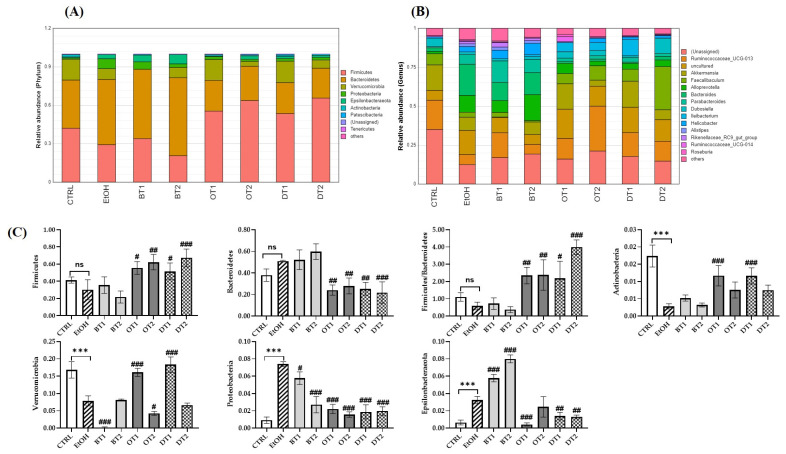

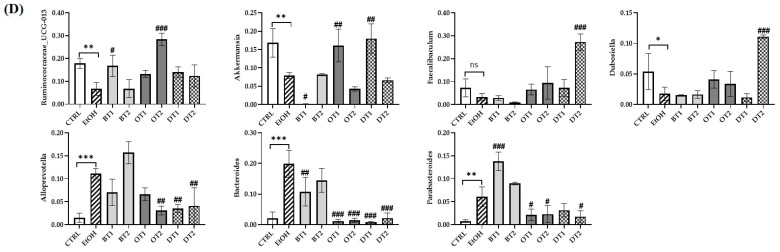
The effects of teas on the composition of microbiota in mice affected by chronic alcohol exposure. CTRL, the control group; EtOH, the model group; BT1, Dianhong Tea; BT2, Yingde Black Tea; OT1, Tieguanyin Tea; OT2, Fenghuang Danzong Tea; DT1, Fu Brick Tea; DT2, Selenium-Enriched Dark Tea. (**A**) Relative abundance of the faecal microbial profile of each group at the phylum level. (**B**) Relative abundance of the faecal microbial profile of each group at the genus level. (**C**) Relative abundance of Firmicutes, Bacteroidetes, the ratio of Firmicutes/Bacteroidetes, Verrucomicrobia, Proteobacteria, Epsilonbacteraeota, and Actinobacteria. (**D**) Relative abundance of Ruminococcaceae_UCG-013, Akkermansia, Faecalibaculum, Alloprevotella, Bacteroides, Parabacteroides, and Dubosiella. * *p* < 0.05, ** *p* < 0.01, *** *p* < 0.001, the model group compared with the control group; # *p* < 0.05, ## *p* < 0.01, ### *p* < 0.001, the tea extract supplementary groups compared with the model group.

It has been demonstrated that the composition of gut microbiota was significantly influenced by chronic alcohol exposure [6]. Our study further identified that the gut microbiota composition was significantly influenced by alcohol consumption and the supplementation of tea extracts. As seen from the Figure 8A,C, at the phylum level, *Firmicutes*, *Bacteroidetes*, *Verrucomicrobia*, *Proteobacteria*, *Epsilonbacteraeota*, *Actinobacteria*, and *Patescilbacteria* were the most abundant bacteria in the fecal samples of all groups (relative abundance > 0.01%). Compared with the control group, chronic alcohol consumption significantly decreased the relative abundance of *Verrucomicrobi* and *Actinobacteria*, and significantly increased the relative abundance of *Proteobacteria* and *Epsilonbacteraeota*. It has been identified that *Proteobacteria* could multiply in the intestine to cope with the imbalance of microbial composition and was closely associated with inflammation [54,55]. In addition, gut microbiota dysbiosis resulted from alcohol was related to an increase in the abundance of *Proteobacteria* [56]. The treatment of Tieguanyin Tea (OT1) and Fu Brick Tea (DT1) extracts significantly inhibited alcohol-induced decrease in *Verrucomicrobi* and *Actinobacteria*, while the treatment of Dianhong Tea (BT1) and Fenghuang Danzong Tea (OT2) extracts further decreased the relative abundance of *Verrucomicrobi*. Additionally, during this experiment, it was found that the increased phylum *Proteobacteria* induced by chronic alcohol consumption was decreased after the intervention of all tea extracts. This result was consistent with the elevated IL-6 and TNF-α levels (caused by alcohol exposure), which were reduced by all tea extracts. This demonstrated that the anti-inflammatory effects of these teas might be associated with their roles in restoring the gut microbiota dysbiosis, which was in agreement with a previous study [54]. Moreover, the supplementation of Tieguanyin Tea (OT1), Fu Brick Tea (DT1), and Selenium-Enriched Dark Tea (DT2) extracts remarkably reduced the relative abundance of *Epsilonbacteraeota*, while Dianhong Tea (BT1) and Yingde Black Tea (BT2) significantly increased it. On the other hand, compared with the control group, an increasing trend in the relative abundance of *Bacteroidetes*, and a decreasing trend in that of *Firmicutes* and the ratio of *Firmicutes/Bacteroidetes* were observed in the model group, but there was no significant difference. However, the treatments of Tieguanyin Tea (OT1), Fenghuang Danzong Tea (OT2), Fu Brick Tea (DT1), and Selenium-Enriched Dark Tea (DT2) extracts significantly decreased the relative abundance of *Bacteroidetes*, and significantly increased that of *Firmicutes* and the ratio of *Firmicutes/Bacteroidetes*.

At the genus level, the relative abundance of *Ruminococcaceae*_UCG-013, *Akkermansia*, and *Dubosiella* was significantly decreased, while that of *Alloprevotella*, *Bacteroides*, and *Parabacteroides* was significantly increased after long-term alcohol exposure (Figure 8B,D). The available evidence suggests that *Akkermansia* is a dominant genus in *Verrucomicrobia* phyla, and plays an essential role in preventing alcohol-induced liver damage by degrading intestinal mucin and improving the gut barrier function [57,58]. A previous study also reported that the abundance of *Akkermansia* was significantly reduced in both mice and humans because of ethanol exposure [59]. Our results found that the abundance of *Verrucomicrobia* and *Akkermansia* was significantly decreased in AFLD mice, and the supplementation of Tieguanyin Tea (OT1) and Fu Brick Tea (DT1) dramatically elevated their abundance. Additionally, the relative abundance of *Ruminococcaceae*_UCG-013 was increased in Dianhong Tea (BT1) and Fenghuang Danzong Tea (OT2) groups, but that of *Akkermansia* was further decreased in Dianhong Tea (BT1) group. Moreover, the relative abundance of *Faecalibaculum* and *Dubosiella* was significantly increased in Selenium-Enriched Dark Tea (DT2) group compared to the model group. Besides, the increased relative abundance of *Alloprevotella*, *Bacteroides* and *Parabacteroides* induced by chronic alcohol exposure was restored partially by the supplementation of Tieguanyin Tea (OT1), Fenghuang Danzong Tea (OT2), Fu Brick Tea (DT1), and Selenium-Enriched Dark Tea (DT2) extracts. However, the relative abundance of *Parabacteroides* was significantly increased by the treatment of Dianhong Tea (BT1) extract.

**Figure 9:**
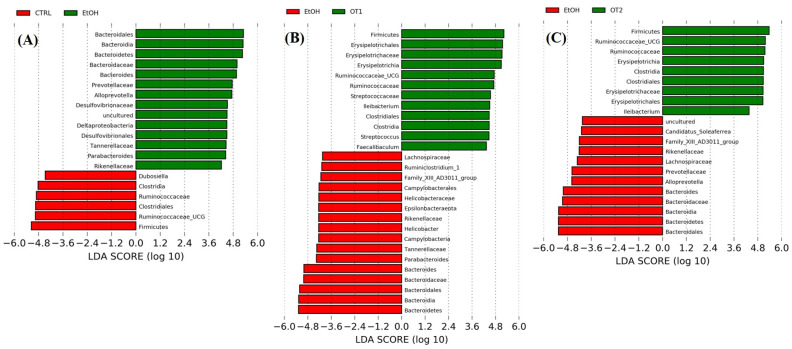

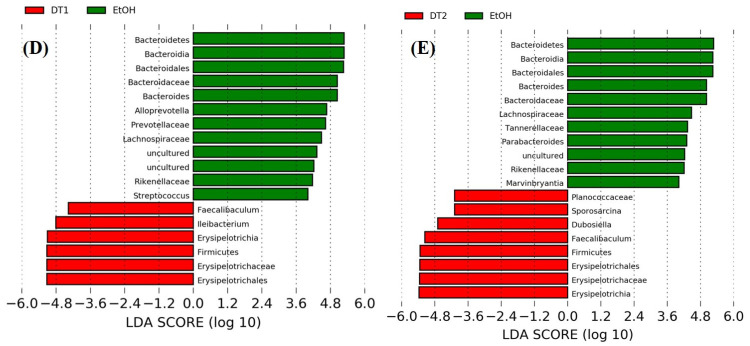
The LDA distribution chart generated from LEfSe showing the most differentially abundant taxa in intestinal microbiota ranging from phylum to genus (LDA score > 4). CTRL, the control group; EtOH, the model group; OT1, Tieguanyin Tea; OT2, Fenghuang Danzong Tea; DT1, Fu Brick Tea; DT2, Selenium-Enriched Dark Tea. (**A**) CTRL (red) + EtOH (green); (**B**) EtOH (red) + OT1 (green); (**C**) EtOH (red) + OT2 (green); (**D**) EtOH (green) + DT1(red); (**E**) EtOH (green) + (red) DT2.

The LEfSe analyses and the cladograms generated according to corresponding LDA scores were also conducted to analyze the most differentially abundant taxa in intestinal microbiota ranging from phylum to genus as shown in Figure 9. Compared with the control group, *Bacteroidetes* phylum, *Bacteroidia* class, *Bacteroidales* order, *Bacteroidaceae* family and *Bacteroides* genus, *Rikenellaceae*, *Prevotellaceae*, and *Tannerellaceae* of the *Bacteroidales* order, *Alloprevotella* of the *Prevotellaceae* family, *Parabacteroides* of the *Tannerellaceae* family, *Deltaproteobacteria* of the *Proteobacteria* phylum, *Desulfovibrionales* of the *Deltaproteobacteria* class, *Desulfovibrionaceae* of the *Desulfovibrionales* order and uncultured microbiota of *Desulfovibrionaceae* family were enriched in the model group. As seen from the Figure 9B–E, the supplementation of Tieguanyin Tea (OT1), Fenghuang Danzong Tea (OT2), Fu Brick Tea (DT1), and Selenium-Enriched Dark Tea (DT2) extracts could mostly prevent chronic alcohol exposure-induced changes in taxa of intestinal microbiota. In addition, the treatments of Tieguanyin Tea (OT1) and Selenium-Enriched Dark Tea (DT2) extracts significantly inhibited the changes in taxa composition of *Tannerellaceae* of the *Bacteroidales* order and *Parabacteroides* of the *Tannerellaceae* family. Moreover, the enrichment in taxa composition of *Alloprevotella* of the *Prevotellaceae* family was significantly reduced in Fenghuang Danzong Tea (OT2) and Fu Brick Tea (DT1) groups. However, the treatments of Dianhong Tea (BT1) and Yingde Black Tea (BT2) did not prevent the changes in taxa of intestinal microbiota in comparison to the model group. Furthermore, it is worth noting that the relative abundance of *Rikenellaceae* family and *Tannerellaceae* family in the gut microbiota of each fecal sample was too low (relative abundance < 0.01%) and might not be able to participate in the regulation of physiological functions. Thus, these results indicated that *Bacteroidetes* phylum, *Bacteroidia* class, *Bacteroidales* order, *Bacteroidaceae* family, and *Bacteroides*, *Alloprevotella*, and *Parabacteroides* genus might be closely associated with alcohol and mainly participated in the initiation and development of AFLD. Similarly, several other studies have identified that alcohol exposure significantly increased the relative abundance of *Bacteroidetes* [13,60]. Moreover, consistent with our study, a previous study reported that the abundance of *Bacteroides* was significantly increased in alcoholic hepatitis mice [53]. The *Bacteroidetes* phylum is composed of three major types of gram-negative bacteria, which have both beneficial and harmful characteristics. An animal study reported that *Bacteroides fragilis* could attenuate colitis by producing polysaccharide A [61]. On the contrary, *Bacteroides* could also induce a pro-carcinogenic effect through producing toxins [62]. This indicated that the intestinal symbiotic bacteria were not absolutely beneficial or harmful. Thus, relative research targeting specific bacteria is needed to clarify their influences in the pathogenesis of AFLD.

### 3.9. The Correlation Analysis between Gut Microbiota and Liver Damage Parameters

**Figure 10:**
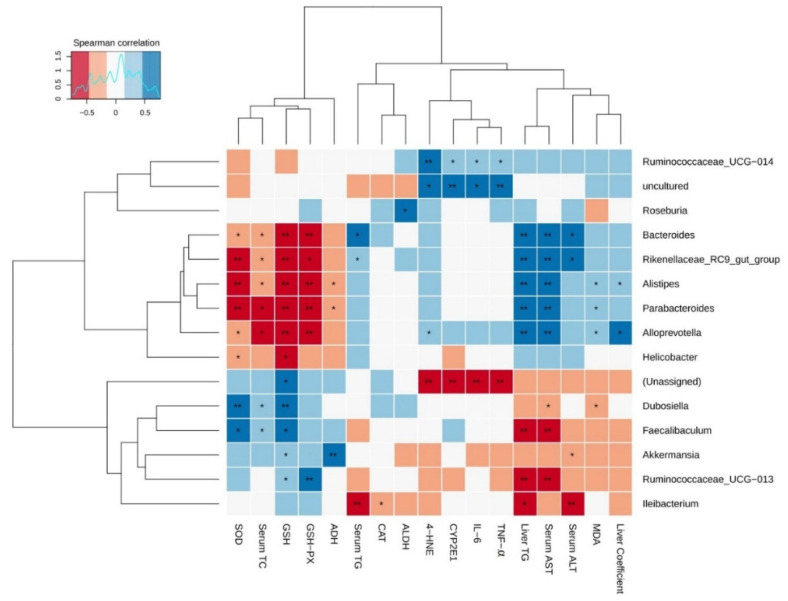
Heat map of the correlation between gut microbiota and liver injury parameters affected by chronic alcohol exposure. The liver damage parameters included hepatic steatosis indicators, serum aminotransferase activity, alcohol metabolism, oxidative stress, and inflammation. Significant difference was represented by asterisk, * *p* < 0.05, ** *p* < 0.01.

To further analyze the correlation between gut microbiota and AFLD induced by chronic alcohol exposure, Spearman’s correlation analysis was employed in the current study. Additionally, there were 15 major gut microbial communities and the relative abundance of each was >0.01%, accounting for more than 90% of each fecal sample. The relationship between these gut microbiota and biochemical indicators of liver injury is presented in the heatmap in Figure 10. Among these bacteria, *Bacteroides*, *Parabacteroides*, *Alloprevotella*, *Alistipes*, *Rikenellaceae*_RC9_gut_group showed significantly positive correlations with serum aminotransferase activities (AST and ALT) and hepatic steatosis (liver coefficient and liver TG content), while *Faecalibaculum*, *Ruminococcaceae*_UCG-013, and *Ileibacterium* showed markedly negative correlations with these parameters. Moreover, weak negative correlations were observed between *Akkermansia* and serum ALT activity, as well as between *Dubosiella* and serum AST activity. Furthermore, *Rikenellaceae*_RC9_gut_group and *Bacteroides* were positively correlated with serum TG, whereas *Ileibacterium* was negatively correlated with it. Furthermore, serum TC was negatively correlated with *Bacteroides*, *Alistipes*, *Parabacteroides*, *Alloprevotella*, and *Rikenellaceae*_RC9_gut_group, and was positively related to *Dubosiella* and *Faecalibaculum*.

The correlation between these major bacteria and alcohol metabolism parameters including CYP2E1, ADH and ALDH was also evaluated. The results showed that uncultured bacteria and *Ruminococcaceae*_UCG-014 were positively correlated with CYP2E1 expression, while unassigned bacteria were negatively correlated with it. In addition, *Roseburia* and *Akkermansia* showed significant positive relationships with ALDH and ADH activities, respectively. Besides, *Alistipes* and *Parabacteroides* showed significantly negative correlations with ADH activity.

Further analysis was performed to analyze the correlations between gut microbiota and biochemical indicators of oxidative stress including antioxidant capacity and lipid peroxidation levels. *Bacteroides*, *Parabacteroides*, *Alloprevotella*, *Alistipes*, and *Rikenellaceae*_RC9_gut_group were negatively associated with the activities of SOD and GSH-Px, as well as GSH content. In addition, *Ruminococcaceae*_UCG-013, *Dubosiella*, *Faecalibaculum*, and *Akkermansia* were positively associated with at least one antioxidant indicators (SOD, GSH-Px or GSH). Moreover, *Helicobacter* showed significant negative correlations with SOD and GSH, and *Ileibacterium* showed an significant negative relationship with CAT activity. For another thing, a significant positive correlation was observed between MDA level and the relative abundance of *Alistipes*, *Parabacteroides*, and *Alloprevotella*, while *Dubosiella* showed an significant positive correlation with MDA level. Furthermore, unassigned bacteria were negatively related to 4-HNE level, whereas *Alloprevotella*, *Ruminococcaceae*_UCG-014 and uncultured bacteria were positively correlated with it. Lastly, the correlations between gut microbiota and liver inflammatory cytokines (IL-6 and TNF-α) revealed that the levels of IL-6 and TNF-α were positively correlated with the relative abundance of *Alloprevotella*, *Ruminococcaceae*_UCG-014 and uncultured bacteria, but negatively associated with that of unassigned bacteria. Taken together, these results indicated that Tieguanyin Tea (OT1), Fenghuang Danzong Tea (OT2), Fu Brick Tea (DT1), and Selenium-Enriched Dark Tea (DT2) prevented AFLD by regulating gut microbiota.

### 3.10. Phenolic Compounds in Fenghuang Danzong Tea and Selenium-Enriched Dark Tea

**Figure 11:**
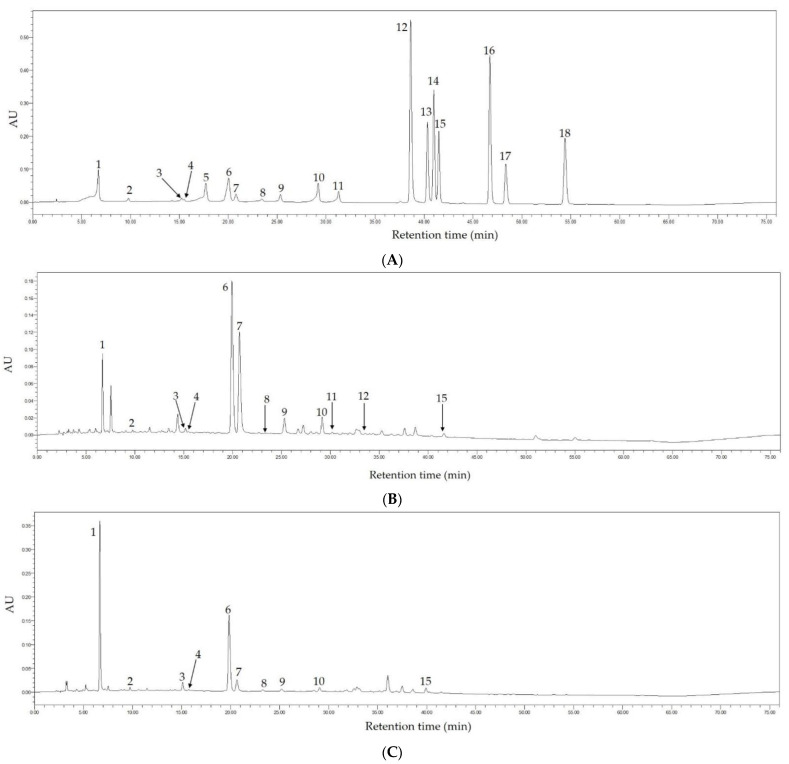
The HPLC chromatograms of the standard compounds (**A**), Fenghuang Danzong Tea (**B**), and Selenium-Enriched Dark Tea (**C**) under 254 nm. 1, gallic acid; 2, gallocatechin; 3, epigallocatechin; 4, catechin; 5, chlorogenic acid; 6, caffeine; 7, epigallocatechin gallate; 8, epicatechin; 9, gallocatechin gallate; 10, epicatechin gallate; 11, catechin gallate; 12, ellagic acid; 13, myricetin; 14, quercitrin; 15, astragalin; 16, quercetin; 17, theaflavin; 18, kaempferol.

In this study, the main phytochemical components in Fenghuang Danzong Tea (OT2) and Selenium-Enriched Dark Tea (DT2) extracts were determined by HPLC because they exerted stronger preventive effects on AFLD caused by chronic alcohol consumption. The chromatograms at 254 nm of the standard compounds, Fenghuang Danzong Tea (OT2) and Selenium-Enriched Dark Tea (DT2) are displayed in Figure 11. In addition, Table 3 shows the main constituents and concentrations in these teas.

Based on the results, 12 and 10 main ingredients have been determined and quantified in Fenghuang Danzong Tea (OT2) and Selenium-Enriched Dark Tea (DT2) extracts, respectively. In addition, it was found that catechins were abundant in teas and epigallocatechin gallate (78.696 ± 1.119 mg/g DW) was the richest catechin in Fenghuang Danzong Tea (OT2), which might be related to antioxidant ability [63]. Moreover, the gallic acid (27.765 ± 0.598 mg/g DW) was the most abundant polyphenol in Selenium-Enriched Dark Tea (DT2), probably due to the degradation of catechins during the fermentation process. For another thing, it was observed that the concentration of caffeine in Fenghuang Danzong Tea (OT2) (27.185 ± 0.316 mg/g DW) and Selenium-Enriched Dark Tea (DT2) (24.284 ± 0.303 mg/g DW) was similar and relatively high, which might be closely related to improving liver steatosis [64].

## 4. Conclusions

In conclusion, the protective effects of black tea, oolong tea and dark tea on AFLD injury mice exposed to chronic alcohol consumption and its regulation of gut microbiota were intensively investigated in the current study. The results showed that the supplementation of tea prevented liver steatosis and inflammation, decreased oxidative stress, and regulated gut microbiota in chronic alcohol-exposed mice, especially the oolong tea and dark tea. However, the black tea showed fewer effects on liver damage caused by chronic alcohol exposure. In addition, the diversity, structure and composition of the intestinal microbiota altered by chronic ethanol exposure could be restored by oolong tea and dark tea supplementation. Moreover, the results indicated that *Bacteroides* could play a potential role in the occurrence and development of AFLD. Furthermore, the findings suggested that *Akkermansia* might be a potential target involved in the protective effects of Tieguanyin Tea (OT1) and Fu Brick Tea (DT1) on AFLD. Thus, targeting gut microbiota might be a promising therapeutic strategy for the prevention or management of AFLD.

## Figures and Tables

**Table 1:** The specific information of six selected teas.

Number	Category	Name	Production Place	Fermentation Degree
BT1	Black Tea	Dianhong Tea	Xishuangbanna, Yunnan	Deep-fermented
BT2	Black Tea	Yingde Black Tea	Yingde, Guangdong	Deep-fermented
OT1	Oolong Tea	Tieguanyin Tea	Xiamen, Fujian	Semi-fermented
OT2	Oolong Tea	Fenghuang Danzong Tea	Shantou, Guangdong	Semi-fermented
DT1	Dark Tea	Fuzhuan Brick tea	Changsha, Hunan	Post-fermented
DT2	Dark Tea	Selenium-Enriched Dark Tea	Enshi, Hubei	Post-fermented

**Table 2:** The effects of teas on gut microbial community richness and diversity.

Groups	Chao 1	Simpson	Shannon
CTRL	11.33 ± 1.70	0.23 ± 0.02	0.78 ± 0.04
EtOH	27.83 ± 2.62 ***	0.12 ± 0.00 ***	1.02 ± 0.01 ***
BT1	18.97 ± 3.64	0.14 ± 0.02	1.02 ± 0.03
BT2	19.23 ± 5.26	0.13 ± 0.01	0.97 ± 0.04
OT1	16.83 ± 2.49 #	0.21 ± 0.02 ##	0.95 ± 0.02
OT2	17.83 ± 1.55 #	0.22 ± 0.03 ##	0.87 ± 0.05 ##
DT1	17.03 ± 2.90 #	0.23 ± 0.04 ###	0.91 ± 0.04 #
DT2	12.17 ± 2.72 ###	0.32 ± 0.02 ###	0.86 ± 0.04 ##

Note: CTRL, the control group; EtOH, the model group; BT1, Dianhong Tea; BT2, Yingde Black Tea; OT1, Tieguanyin Tea; OT2, Fenghuang Danzong Tea; DT1, Fu Brick Tea; DT2, Selenium-Enriched Dark Tea. Chao 1 reflects community richness of intestinal flora; Simpson and Shannon reflect community diversity of intestinal flora. *** *p* < 0.001, the model group compared with the control group; # *p* < 0.05, ## *p* < 0.01, ### *p* < 0.001, the tea extract supplementary groups compared with the model group.

**Table 3:** The contents (mg/g DW) of main phytochemicals in Fenghuang Danzong and Selenium-Enriched Dark Tea.

Main Phytochemicals	Fenghuang Danzong Tea	Selenium-Enriched Dark Tea
gallic acid	7.661 ± 0.313	27.765 ± 0.598
gallocatechin	2.732 ± 0.0795	7.950 ± 0.135
epigallocatechin	4.424 ± 0.0221	14.140 ± 0.436
catechin	2.672 ± 0.065	3.001 ± 0.032
chlorogenic acid	-	-
caffeine	27.185 ± 0.316	24.284 ± 0.303
epigallocatechin gallate	78.696 ± 1.119	15.441 ± 0.428
epicatechin	1.609 ± 0.013	4.261 ± 0.094
gallocatechin gallate	11.499 ± 0.224	4.980 ± 0.207
epicatechin gallate	3.954 ± 0.085	1.585 ± 0.033
catechin gallate	0.816 ± 0.011	-
ellagic acid	0.408 ± 0.021	-
myricetin	-	-
quercitrin	-	-
astragalin	0.365 ± 0.006	0.794 ± 0.101
quercetin	-	-
theaflavin	-	-
kaempferol	-	-

Note: -, not determined; DW, dry weight of tea.

## Data Availability

The data is kept in School of Public Health, Sun Yat-Sen University.
